# Cryptococcal Osteomyelitis in an Immunocompetent Patient

**DOI:** 10.7759/cureus.21074

**Published:** 2022-01-10

**Authors:** Andrea Dumenigo, Mitali Sen

**Affiliations:** 1 Internal Medicine, Philadelphia College of Osteopathic Medicine, Philadelphia, USA; 2 Rheumatology, Einstein Medical Center Philadelphia, Philadelphia, USA

**Keywords:** osteomyelitis, septic arthritis, disseminated cryptococcosis, cryptococcosis, immunocompetent patients, cryptococcus neoformans (c. neoformans)

## Abstract

Cryptococcal osteomyelitis is extremely rare. When cryptococcal infections occur, they are usually caused by *Cryptococcus neoformans* and involve the lungs or central nervous system in an immunocompromised individual. However, we report a case of osteomyelitis of the right ankle and right elbow due to *C. neoformans* in a 29-year-old immunocompetent male.

## Introduction

*Cryptococcus neoformans* is an opportunistic fungus that usually affects the pulmonary and central nervous systems of immunocompromised individuals [1]. Bone involvement as the presenting manifestation is scarcely found in the current literature. Incidence of bone involvement is between 5% and 10% and is most commonly due to hematogenous seeding from a pulmonary focus or a lymph node [2]. *C. neoformans* infections are usually seen in patients with human immunodeficiency virus (HIV) infection or patients who have undergone an organ transplant [3].

This report presents a case of a 29-year-old male, with no comorbidities, who presented to the hospital with chronic oligoarticular joint pain and swelling and was subsequently diagnosed with cryptococcal osteomyelitis in two non-contiguous joints.

## Case presentation

A 29-year-old Caucasian male with no medical history presented with right elbow and right ankle pain and swelling, which led to decreased mobility of the elbow and decreased weight bearing on the ankle. He stated that these symptoms started about four months ago and were progressively getting worse. He visited his primary care physician a week prior and was treated empirically with one week of sulfamethoxazole-trimethoprim 800 mg and cephalexin 500 mg for suspected skin and subcutaneous infection with a slight improvement in erythema.

He denied any preceding symptoms of fever, diarrhea, cough, chest pain, dyspnea, palpitations, or skin rashes. He also denied any significant family history. He was a former smoker and admitted to occasional inhalational and edible marijuana use. The patient denied illicit IV drug use, alcohol use, incarceration, or travel outside of the United States within the past year. The patient worked from home as a software engineer. He had recently moved into a new house and had begun working on renovating the basement. He also reported that he used to smoke away from his home, close to an old house that had a bird feeder in it.

On presentation, he was hemodynamically stable and afebrile. His examination was significant for a soft tissue tender erythematous swelling on the right elbow and right ankle. He was also noted to have a 2 x 2 cm open wound on the right lower extremity close to the ankle, which was exuding purulent fluid with pressure. The joint examination was also significant for decreased range of movements of the ankle and elbow without any synovitis in any other joint. The remainder of the physical examination including lung examination was normal.

His laboratory workup was significant for elevated erythrocyte sedimentation rate (ESR) of 43 mm/hr (reference range: 0-22 mm/hr) and C-reactive protein (CRP) level of 29.5 mg/L (0-10 mg/L). White blood cell count was 7.7 x 10^3^/mcL (reference range: 4.5 -11.0 x 10^3^/mcL) with a normal lymphocyte count. His HIV test results came back negative.

**Figure 1:**
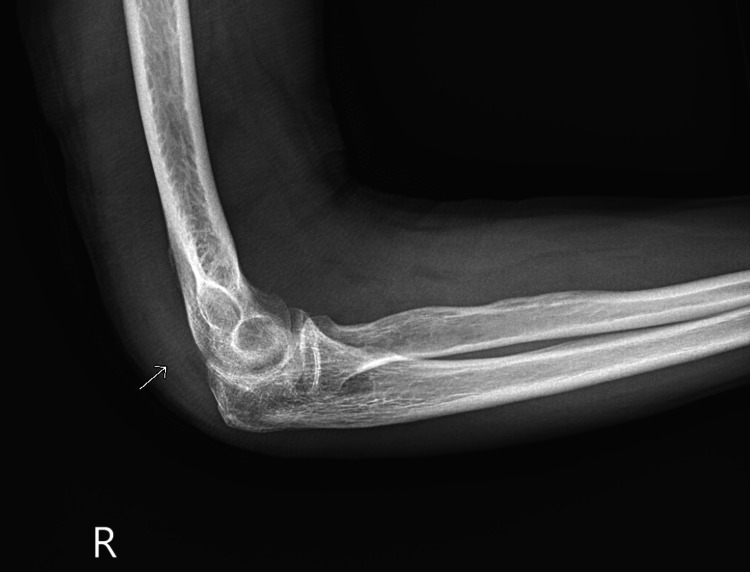
X-ray of the right elbow. Large joint effusion distending the anterior recess. There is a periosteal reaction in the posterior aspect of the humerus and erosions in the coronoid fossa of the distal humerus, creating a scalloped appearance. There is preservation of the joint space and mild periarticular osteopenia.

**Figure 2:**
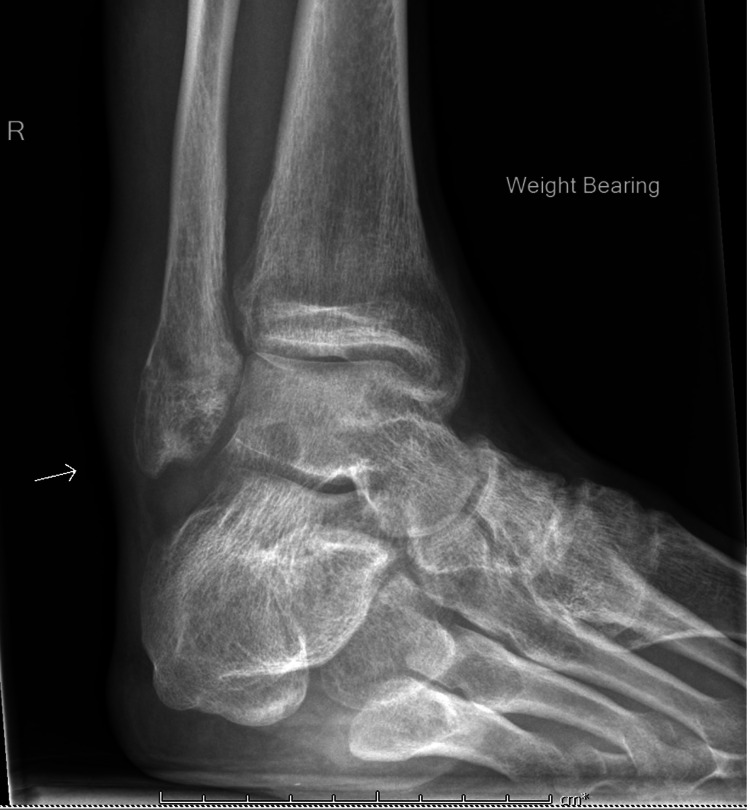
X-ray of the right ankle. Marked soft tissue swelling around the right ankle with marked periarticular osteopenia. There is also a lobulated soft tissue density projecting dorsally to the ankle joint into Kager's fat pad. There is preservation of the joint space width.

**Figure 3:**
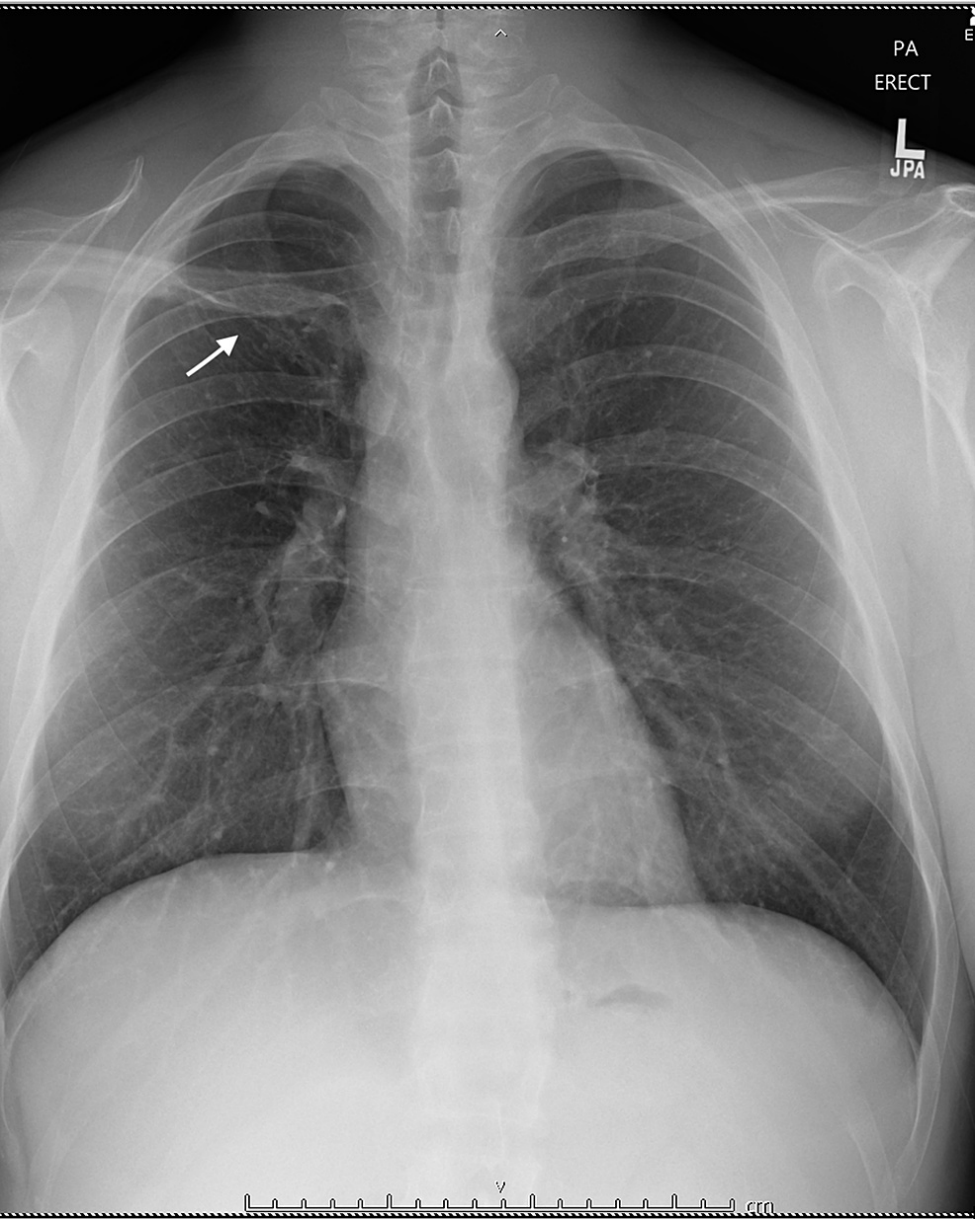
Chest X-ray. Linear scarring or subsegmental atelectasis in the right upper lobe.

**Figure 4:**
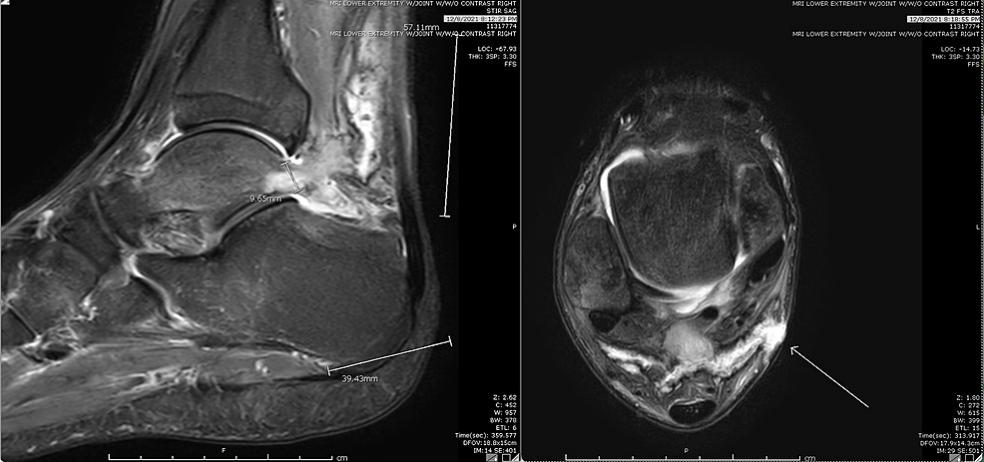
MRI of the lower extremity. A small open wound at the posteromedial aspect of the ankle with a subjacent complex rim enhancing fluid collection concerning for an abscess measuring 1.4 x 1.5 x 1.0 cm is noted. There is intraosseous penetration of this complex collection with associated erosion involving the lateral tubercle of the posterior talar process, although there is no surrounding T1 hypointense marrow replacement. There is somewhat more focally pronounced surrounding bone marrow edema-like signal noted throughout the talus as well as within the posterior subtalar facet of the calcaneus extending into the posterior superior calcaneal body, amidst a background of diffuse periarticular hyperemia/osteopenia throughout the ankle, hindfoot, and midfoot, with associated small effusions and synovitis of the ankle and posterior subtalar joints.

Imaging of the right elbow and right ankle revealed diffuse osteopenia, effusion, and periarticular erosion (Figures 1, 2). Chest X-ray showed linear scarring or subsegmental atelectasis in the right upper lobe (Figure 3). MRI of his right lower extremity showed a small open wound at the posteromedial aspect of the ankle with a subjacent complex rim-enhancing fluid collection with intraosseous penetration of this complex collection with associated erosion involving the lateral tubercle of the posterior talar process. Focally pronounced surrounding bone marrow edema was noted throughout the talus amidst a background of diffuse periarticular hyperemia/osteopenia throughout the ankle, hindfoot, and midfoot, with associated small effusions and synovitis of the ankle and posterior subtalar joints (Figure 4).

The patient was taken to the operating room where he underwent wound debridement and joint irrigation of his right elbow and right ankle. Intra-operatively, significant purulence and necrotic tissue were noted in both joints. Preliminary bacterial, fungal, and AFB stains were all negative. On the third day, a few yeasts were seen on the sample from the right elbow and right ankle, and over the next week, the synovial fluid cultures grew *Cryptococcus neoformans*. Blood cultures were sterile and serum cryptococcal antigen was negative. He was started on oral fluconazole 800 mg daily for two weeks with significant improvement in pain and swelling. He also started physical therapy and is now back to functional baseline with a plan to complete oral fluconazole 400 mg daily for six months.

## Discussion

Cryptococcal articular disease is caused by the basidiomycetous yeasts *Cryptococcus neoformans* or by *Cryptococcus gattii* [4]. In the United States, cryptococcosis incidence is decreasing due to antiretroviral therapy for HIV patients, and mortality is about 12% [4]. *C. neoformans* is more prevalent and can be found in soil or pigeon excreta [5]. Cryptococcus fungal infections are mostly seen in immunocompromised patients but can very rarely present in immunocompetent patients. Most commonly, the cryptococcal disease affects an immunocompromised patient's lungs or central nervous system. Few HIV-negative patients have been reported to have cryptococcal osteomyelitis [2]. These patients had predisposing factors such as sarcoidosis, tuberculosis, steroid therapy, lymphoma, leukemia, Hodgkin’s disease, and diabetes mellitus [2].

Cryptococcal osteomyelitis is usually caused by hematogenous seeding, and less commonly by direct inoculation of the organism via the skin after trauma [2,3]. Of the reported cases of cryptococcal osteomyelitis, the most common site of disease is the vertebrae [3].

Our case describes a 29-year-old immunocompetent male who presented with cryptococcal osteomyelitis. This case is unique because of the negative serum cryptococcal antigen despite cultures being positive. No other reported cases have shown non-contiguous joint involvement, specifically of ipsilateral elbow and ankle, both of which are rarely involved in septic arthritis.

It can be postulated that the patient's infection most likely occurred due to the patient's exposure to pigeon excreta due to him regularly smoking close to a bird feeder and started with asymptomatic lung involvement, then had intermittent fungemia leading to seeding of the affected joints.

There is limited information regarding the therapy of cryptococcal osteomyelitis in immunocompetent patients due to how rare the disease is. According to the current guidelines of the Infectious Diseases Society of America (IDSA), non-meningeal, non-pulmonary cryptococcosis should be treated with fluconazole 400 mg daily for six to 12 months [6].

## Conclusions

We discussed a rare case of cryptococcal osteomyelitis presenting as oligoarticular joint involvement in an immunocompetent patient affecting noncontiguous joints. This infection is mostly seen in immunocompromised individuals, specifically those that are HIV positive. Through this case vignette, we would like to bring the clinicians' attention to this organism as an extremely rare cause of osteomyelitis in immunocompetent patients, as well as treatment options in this situation.
